# Supplementary material for: Sterol derivative binding to the orthosteric site causes conformational changes in an invertebrate Cys-loop receptor
Source: eLife. 2023 Jul 3;12:e86029. doi: 10.7554/eLife.86029 (PMC10338036; doi:10.7554/eLife.86029)
Supplement: Supplementary file 1. — Ion channel PDB identifiers, channel conformation, ligands, and narrowest constriction residues are listed. [file elife-86029-supp1.docx]

| **Cationic**  **channel** | **PDB** | **State** | **Minimal diameter (Å)** | **Constriction Residue(s)** | **Position** | **Ligand** | **Detergent / nano disck** | **Added lipids** |
| --- | --- | --- | --- | --- | --- | --- | --- | --- |
| Alpo4 |  | Closed | ∼2.4 | Met265 | 16' | - | LMNG |  |
|  |  | Desensitized | ∼2.1 | Met265 | 16' | CHAPS | LMNG, CHAPS |  |
| mouse 5-HT3A | 6HIN | Open | ∼6.0 | Ser253 | 2' | Serotonin | Detergent |  |
|  | 6HIO | Pre-wet | ∼4.4 | Leu260 | 9' | Serotonin | Detergent |  |
|  | 6HIQ | Desensitized | ∼5.0 | Ser253 | 2' | TMPPAA | Detergent |  |
|  | 6HIS | Closed | ∼4.4 | Glu250 | -1' | Tropisetron | Detergent |  |
| human α4β2 | 5KXI_A | Desensitized | ∼3.8 | Glu | -1' | Nicotine | Detergent |  |
|  | 5KXI_B |  |  |  |  |  |  |  |
| human α3β4 | 6PV7_A | Desensitized | ∼1.7 | Glu | -1' | Nicotine | Saposin A |  |
|  | 6PV7_B |  |  |  |  |  |  |  |
|  | 6PV8_A | Desensitized | ∼1.7 | Glu | -1' | AT-1001 | DDM |  |
|  | 6PV8_B |  |  |  |  |  |  |  |
| human α7 | 7KOO | Resting | ∼2.4 | Leu247 | 9' | α-bungarotoxin | 1 mM DDM | 10 uM SBL , 0.25% Cholesterol |
|  | 7KOX | Activated | ∼7.2 |  |  | Epi + PNU |  |  |
|  | 7KOQ | Desensitized | ∼4.3 | Glu237 | -1' | Epibatidine |  |  |
| torpedo αγαδβ | 7QKO | Resting | ∼2.0 | Leu258 | 16' | - | MSP2N2 | NaCholate, SBL |
|  | 7QL6 | Desensitized | ∼6.0 | Thr, Ser244 | 2' | Carbachol |  |  |
|  | 7QL5 |  |  |  |  | Nicotine |  |  |
|  | 7SMM | Resting | ∼2.5 | Leu258 | 16' | - | Saposin A | Cholesterol, SBL (1:4) |
|  | 7SMQ |  |  |  |  | Apo with cholesterol |  |  |
|  | 7SMR | Desensitized | ∼4.5 | Thr, Ser244 | 2' | Carbachol |  |  |
|  | 7SMS |  |  |  |  | d-Turbo |  |  |
|  | 7SMT |  |  |  |  | d-Turbo, carb |  |  |
| ELIC | 6HJX | Closed | ∼1.3 | Phe247 | 16' | - | UDM |  |
|  | 8D64 | Unassigned | <2.0 | Phe247 | 16' | Cysteamine | MSP1E3D1 | POPC |
|  | 8D63 | Resting | <1.0 |  |  | - |  |  |
|  | 8D66 | Unassigned | <2.0 |  |  | Cysteamine |  | 2POPC |
|  | 8D65 | Resting | <1.0 |  |  | - |  | 1POPE |
| ELIC3 | 8D67 | Unassigned | <2.0 | Leu240 | 9' | Cysteamine |  | 1POPG |
| ELIC5 | 8D68 | Open | ∼12.6 | - | - | Cysteamine |  |  |
| ELIC | 8F32 | Pre-active |  | Phe247 | 16' | Cysteamine | SMA |  |
|  | 8733 | Pre-active |  |  |  |  | Saposin A | 2POPC |
|  | 8F34 | Desensitized | ∼3.0 |  | 2' |  | spMSP1D1 | 1POPE |
|  | 8F35 | Closed |  |  | 16' | - | spMSP1D1 | 1POPG |
| GLIC | 4HFI |  |  | Thr226 | 2' | DDM |  |  |
|  |  |  |  |  |  |  |  |  |
