# Supplementary material for: Sterol derivative binding to the orthosteric site causes conformational changes in an invertebrate Cys-loop receptor
Source: eLife. 2023 Jul 3;12:e86029. doi: 10.7554/eLife.86029 (PMC10338036; doi:10.7554/eLife.86029)
Supplement: Supplementary file 2. — Name of the compounds, their CID number, and average binding energies across the top five docking poses are shown. Binding energies for acetylcholine, GABA, and glycine are shown for comparison. [file elife-86029-supp2.docx]

| Compound | CID | Average Binding energy (kcal/mol) | Theoretical Kd (nM)* |
| --- | --- | --- | --- |
| Bemcentinib (R428) | 46215462 | -12.74 | 0.45 |
| Adapalene | 60164 | -12.34 | 0.88 |
| Diosgenin | [99474](https://pubchem.ncbi.nlm.nih.gov/compound/99474) | -12.1 | 1.3 |
| ZINC36126889 | [93172717](https://pubchem.ncbi.nlm.nih.gov/compound/93172717) | -12.08 | 1.4 |
| Metagenin | 12312773 | -11.3 | 5.1 |
| Proscillaridin | 5284613 | -11.16 | 6.5 |
| CHEMS | 65082 | -10.52 | 19 |
| CHAPS | [107670](https://pubchem.ncbi.nlm.nih.gov/compound/107670) | -9.5 | 107 |
| CHAPSO | [122145](https://pubchem.ncbi.nlm.nih.gov/compound/122145) | -9.2 | 178 |
| ACh | 187 | -4.04 | 1.1 10^6^ |
| GABA | 119 | -3.92 | 1.3 10^6^ |
| Glycine | 750 | -3.6 | 2.3 10^6^ |

***** The dissociation constant, K_d,_ is calculated as K_d_=exp(ΔG/RT), where R=8.31 J/mol•K and T=298 K.
